# Biomimic Nanodrugs Overcome Tumor Immunosuppressive Microenvironment to Enhance Cuproptosis/Chemodynamic‐Induced Cancer Immunotherapy

**DOI:** 10.1002/advs.202411122

**Published:** 2024-12-12

**Authors:** Hangyi Wu, Xiaoyu Lu, Yuhan Hu, J. Baatarbolat, Zhihao Zhang, Yiping Liang, Youwen Zhang, Ye Liu, Huixia Lv, Xin Jin

**Affiliations:** ^1^ Department of Pharmaceutics China Pharmaceutical University Nanjing Jiangsu 211198 China; ^2^ Phase I clinical trial center The Affiliated Suzhou Hospital of Nanjing Medical University Suzhou Jiangsu 215000 China; ^3^ Department of Pharmaceutics The affiliated Suqian First People's Hospital of Nanjing Medical University Suqian Jiangsu 223800 China

**Keywords:** chemodynamic therapy, cuproptosis, elesclomol, immunogenic cell death, membrane coating, NLG919, tumor immunosuppressive microenvironment

## Abstract

Elesclomol (ES) as an efficient Cu ionophore can specifically transport Cu into mitochondria and disrupt intracellular Cu homeostasis. Extra intracellular Cu induces cuproptosis and chemodynamic therapy (CDT), which further cascades immunogenic cell death (ICD) and activates antitumor immune responses. However, the tumor immunosuppressive microenvironment (TIM) attenuates the efficiency of the immune response. Herein, a biomimic nanodrug (ECNM) is fabricated, of which ES, Cu^2+^ and NLG919 (an IDO1 inhibitor) are integrated via a self‐assembly process and subsequently coated with 4T1 cell membrane. ECNM can overcome the typical drawbacks of ES, ameliorating the stability and half‐life of ES by membrane‐coating and enhancing its tumor accumulation and internalization via homotypic targeting. It is worth mentioning that, the addition of NLG919 is also beneficial to the system circulation stability of ES and reduces the non‐specific ES release. After internalization, ECNM dissociates via the glutathione‐responsive process and exhibits comprehensive antitumor capabilities, including cuproptosis, CDT and TIM reversing, thereby eliciting ICD and optimizing the antitumor immune response. Furthermore, ECNM not only accelerates tumor regression but also gains a strong abscopal effect and displays the potential of tumor vaccination. Overall, ECNM can activate antitumor immunity via cuproptosis and CDT, together with TIM reversing, for cancer treatment.

## Graphical abstract

In this study, a biomimic nanodrug of 4T1 cell membrane‐camouflaged ES/Cu^2+^/NLG919 self‐assembly chelate (ECNM) is developed. ECNM can activate antitumor immunity via cuproptosis and CDT, together with TIM reversing, for cancer treatment.

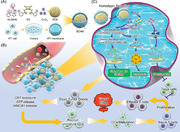

## 1 Introduction

Copper is an essential trace element in the cellular metabolic process. Recently, a Cu‐dependent cell death pathway, “cuproptosis”, was first defined and reported by Tsvetkov et al. and rapidly became a research hotspot.^[^ ^1^, ^2^, ^3^ ^]^ Unlike other cell death pathways, such as necroptosis, pyroptosis and ferroptosis, cuproptosis is characterized by intracellular Cu overload, which induces robust cellular dysfunctions. Ferredoxin‐1 (FDX1), as an upstream regulator of protein lipoylation, plays a vital role in cuproptosis. It promotes tricarboxylic acid recycle‐associated enzyme lipoylation and causes proteotoxic stress for cell death.^[^ ^1^, ^2^, ^3^, ^4^ ^]^ However, owing to the special transportation and balancing mechanisms, intracellular Cu is far from triggering cuproptosis.^[^ ^5^, ^6^ ^]^ To address this issue, copper ionophores are mentioned for Cu import. Among them, ES as the “founding partner” of cuproptosis is seriously recommended due to its high biosafety and mitochondria‐targeting transportation capacity.^[^ ^3^, ^7^ ^]^ In addition to cuproptosis, the enhanced intracellular Cu can also induce CDT, of which Cu^2+^ reacts with glutathione (GSH), and the reduced Cu^+^ together with H_2_O_2_ further generates highly toxic hydroxyl radical (·OH).^[^ ^8^, ^9^ ^]^ It is worth mentioning that, chemodynamic therapy (CDT) rather than cuproptosis was the actual initial research and development significance of ES.^[^ ^10^, ^11^ ^]^ Unfortunately, the clinical application of ES was interrupted because of its unsatisfactory system circulation stability, low half‐life and insufficient tumor accumulation.^[^ ^12^, ^13^ ^]^

Recently, hundreds of studies have focused on Cu transport for cuproptosis and CDT, and almost all of them demonstrated the presence of the cascade immunogenic cell death (ICD) and immune response at the end of their research.^[^ ^14^, ^15^, ^16^, ^17^ ^]^ For the ICD cascade, tumor cells release tumor‐associated antigens (TAAs) and damage‐associated molecular patterns (DAMPs), which in turn recruit and regulate dendritic cells (DCs) for antigen‐presenting.^[^ ^14^, ^18^ ^]^ Then, the matured DCs activate the proliferation and differentiation of naïve T cells to effector T cells, ultimately stimulating antitumor immune response.^[^ ^3^, ^19^ ^]^ Essentially, intravenous administration of the cuproptosis/CDT activator (intracellular effective drugs) is effective against the matured and leaky tumor tissues and merely works after internalization, while the suppression of inaccessible migration and recurrence tissues is mainly attributed to the cascade ICD and immune responses.^[^ ^20^, ^21^ ^]^ Therefore, the induced ICD and immune process are worthwhile. However, the presence of TIM commonly hinders the function of immune cells.^[^ ^22^, ^23^ ^]^ In solid tumors, endogenous indoleamine 2,3‐dioxygenase 1(IDO1) is highly expressed and involved in tumor immunosuppressive microenvironment (TIM).^[^ ^24^, ^25^ ^]^ The overexpressed IDO1 can catalyze the decomposition of tryptophan (Trp) to kynurenine (Kyn). Trp exhaustion reduces a major “energy” for cytotoxic T lymphocytes, and Kyn elevation recruits more regulatory T cells (T_regs_) which is a primary component for TIMs. Ultimately, the above process causes immune escape and the failure of cancer immunotherapy.^[^ ^26^, ^27^ ^]^ Therefore, copper‐induced cuproptosis/CDT in combination with TIM reversal is a potential strategy for cancer treatment.

**Scheme 1:**
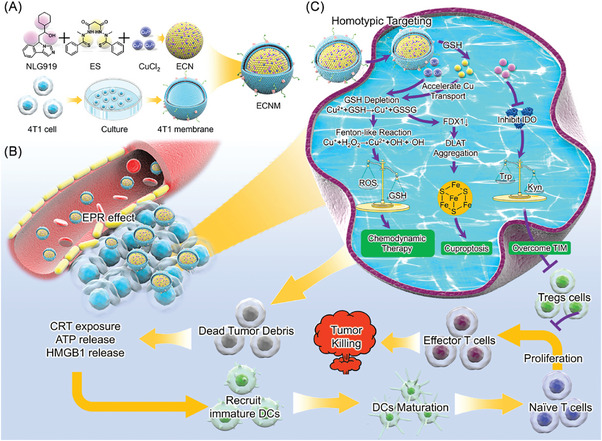
A) Schematic illustration of ECNM fabrication. B) ECNM accumulated in tumor sites via the EPR effect. C) ECNM could be internalized into the cytoplasm by homotypic targeting and induce the synergies of cuproptosis, CDT and TIM reversing, which could activate T cell‐mediated immune response for cancer immunotherapy.

In this study, NLG919, an IDO1 highly selective inhibitor, is utilized to reverse TIM and amplify ES‐Cu (EC)‐related antitumor immunity. However, the application of NLG919 is constrained by unsatisfied solubility and bioavailability.^[^ ^28^, ^29^ ^]^ To solve this problem, we directly induced NLG919 into EC. As displayed in **Scheme** **1**, owing to the unsaturated aromatic ring and lone pair electron of NLG919, we hypothesized that NLG919 could self‐assemble with ES and Cu^2+^ into uniform nanoparticles (denoted as ECN) via coordination interaction and π‐π stacking. ECN was considered to enhance the solubility of both NLG919 and ES. It is worth mentioning that NLG919 was reported to improve the stability of nanoparticles by enhancing the interaction force,^[^ ^30^, ^31^ ^]^ which was able to reduce the non‐specific drug release in the system circulation, making the combination of NLG919 and EC a natural fit. Then, membrane decoration is applied to further address the typical drawbacks of ES, because it can not only ameliorate the system circulation stability and half‐life of the nanoparticles via the immune camouflage effect but also enhance their tumor accumulation and internalization via homotypic targeting.^[^ ^32^, ^33^ ^]^ For the reasons mentioned above, a biomimic nanodrug (ECNM) was fabricated, of which ES, Cu^2+^ and NLG919 were integrated via a self‐assembly process and subsequently coated with the 4T1 cell membrane. Then, ECNM was systematically characterized and detected in vitro and in vivo. The results showed that ECNM could effectively enhance the system circulation half‐life and tumor accumulation of ES after intravenous administration. After internalization, ECNM released ES and NLG919 via GSH‐mediated dissociation due to the high affinity between GSH and Cu^2+^. On one side, ES could transport extracellular Cu into the cytoplasm to exert Cu overload, which triggered cuproptosis and CDT. Dying tumor cells under cuproptosis and CDT elevated calreticulin (CRT) exposure and the release of adenosine triphosphate (ATP) and high mobility group protein (HMGB1), which activated DC proliferation and maturation and the cascade T cell immune response. On the other side, NLG919 reversed the TIM by downregulating the expression of IDO1 and inhibited T cell exhaustion, amplifying the antitumor immune response. Finally, ECNM effectively accelerated the tumor regression and displayed superior abscopal effect and tumor vaccination potential. Overall, ECNM is an attractive strategy for tumor immunotherapy because of the synergies of the cuproptosis/CDT process and TIM reversing.

## 2 Results and Discussions

### 2.1 Characterization of ECNM

**Figure 1:**
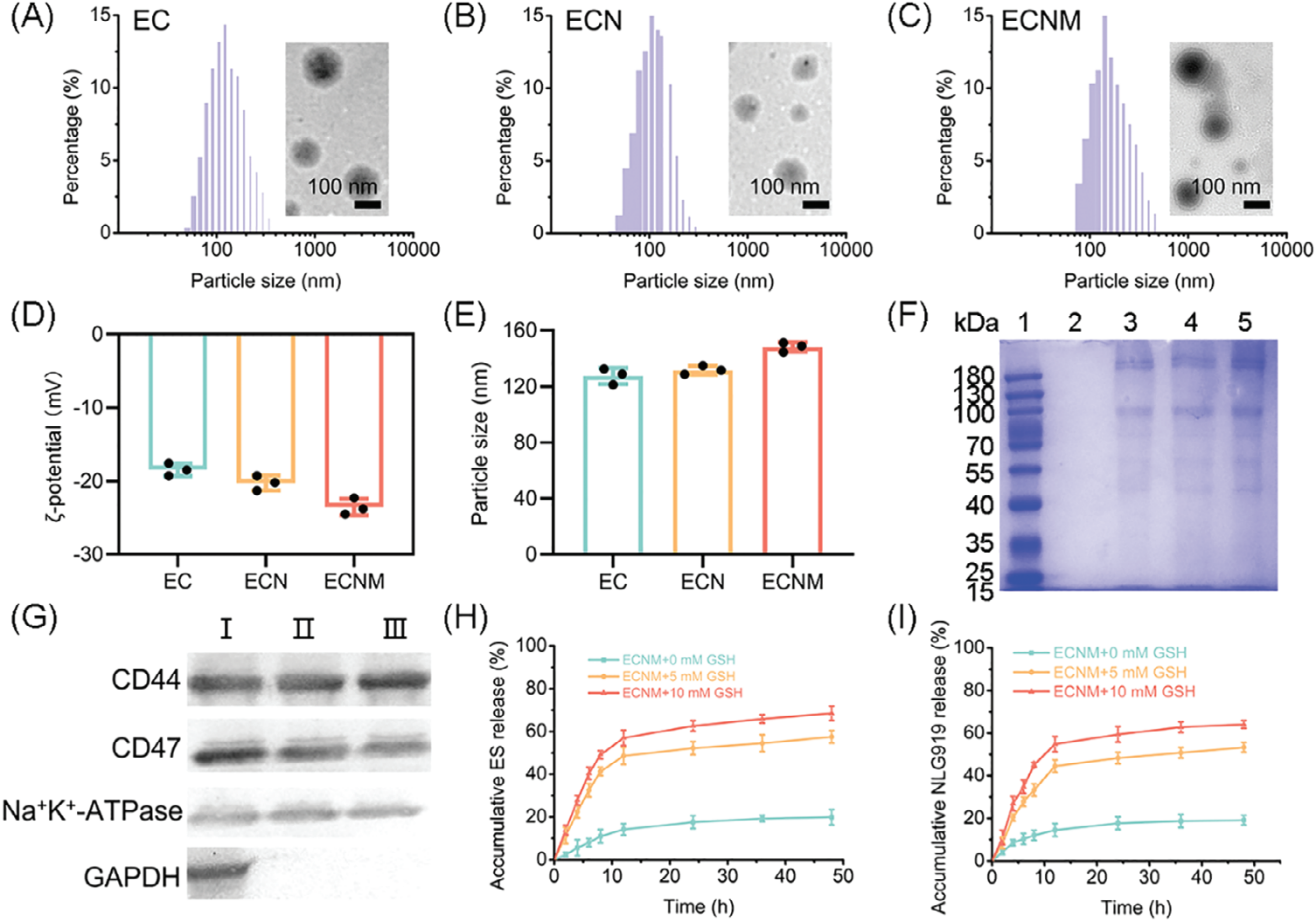
Characterization of ECNM. A–C) Particle size distribution and TEM images of EC, ECN and ECNM. Scale bar: 100 nm. D–E) ζ‐potential and particle size of the nanoparticles. F) SDS‐PAGE assay of ECNM. Band 1, 2, 3, 4, and 5 represented marker, ECN, 4T1 cell membrane, 4T1 cell membrane/ECN mixture and ECNM, respectively. G) Membrane markers detection by Western blot. I, II and III represented 4T1 cell, 4T1 cell membrane and ECNM, respectively. H,I) Accumulative drug release of ES and NLG919 from ECNM with/without GSH at 37 °C, *n* = 3. Data are presented as mean ± SD.

ECNM was fabricated into two steps, including the preparation of ECN and 4T1 cell membrane decoration. ECN was formed by self‐assembly of ES, NLG919 and Cu^2+^ via coordination force between ES and Cu^2+^ and π‐π stacking among the lone pair electrons and aromatic rings of ES and NLG919. The cell membrane was camouflaged on the surface of ECN via the sonication method. EC was composed of ES and Cu^2+^ without NLG919.^[^ ^3^, ^34^ ^]^ The encapsulation efficacy (EE, %) and loading content (LC, %) of ES and NLG919 were measured as reflected in Table S1 (Supporting Information). Owing to the carrier‐free characteristics of both EC, ECN and ECNM, all nanoparticles displayed a high loading content of the therapeutic agents. As shown in **Figure** **1**A–C, the nanoparticles were well dispersed and gained a uniform particle size distribution. TEM images revealed that EC and ECN had a solid spherical‐like morphology, and ECNM displayed an extra cell membrane layer. The ζ‐potential and particle size were both measured. As shown in Figure 1D,E, EC and ECN showed a negative surface potential (≈‐20 mV) and a favorable particle size (≈130 nm). As for ECNM, both ζ‐potential and particle size were slightly increased after membrane coating. Then, SDS‐PAGE was applied to confirm the presence of the 4T1 cell membrane. As shown in Figure 1F, ECNM retained most membrane intrinsic protein. In addition, cell membrane markers were measured by Western blot (Figure 1G). It showed that CD47, CD44 (transmembrane glycoprotein) and Na^+^/K^+^‐ATPase (ion transporter) were all expressed in the 4T1 cell (band I), 4T1 cell membrane (band II) as well as ECNM (band III), while GAPDH could not be observed on bands II and III, indicating the cell membrane was successfully isolated with the integrity protein diversity.

Controlled delivery of antitumor‐active ingredients largely determines the efficacy and biocompatibility. Therefore, condition mediums mimicking the system circulation and tumor sites (high GSH level) were customized to evaluate the drug‐release behavior of ECNM. As shown in Figure 1H,J, in the absence of GSH, only approximately 20% of ES and NLG919 were released from ECNM after 48 h‐incubation, suggesting that ECNM was stable in the system circulation and able to reduce the non‐specificity ES release. However, with the addition of 5 mM GSH, the accumulative drug release increased to about 55% for both NLG919 and ES, and a higher release rate (approximately 65%) was observed with 10 mM GSH, indicating that ECNM could respond to the redox condition and rapidly unload cargoes in the tumor site. The selective drug‐release performance was ascribed to the high affinity between GSH and Cu^2+^.^[^ ^8^, ^9^ ^]^ GSH could competitively bind Cu^2+^ in ECNM and induce the dissociation of the nanocarrier. Then, the storage stability of EC, ECN and ECNM was characterized via particle size. As shown in Figure S1 (Supporting Information), ECNM displayed superior storage stability, while EC and ECN gradually aggregated during 10 day's incubation without membrane‐coating, especially under the condition of 10% FBS. Moreover, compared with ECN, EC seemed to be more prone to aggregation.

### 2.2 Molecular Dynamics Simulation Assay

The formation process of multi‐component self‐assembly systems is often controversial. Herein, a molecular dynamics simulation assay was applied to deduce the self‐assembly of the nanoparticles and explain the potential mechanism.

**Figure 2:**
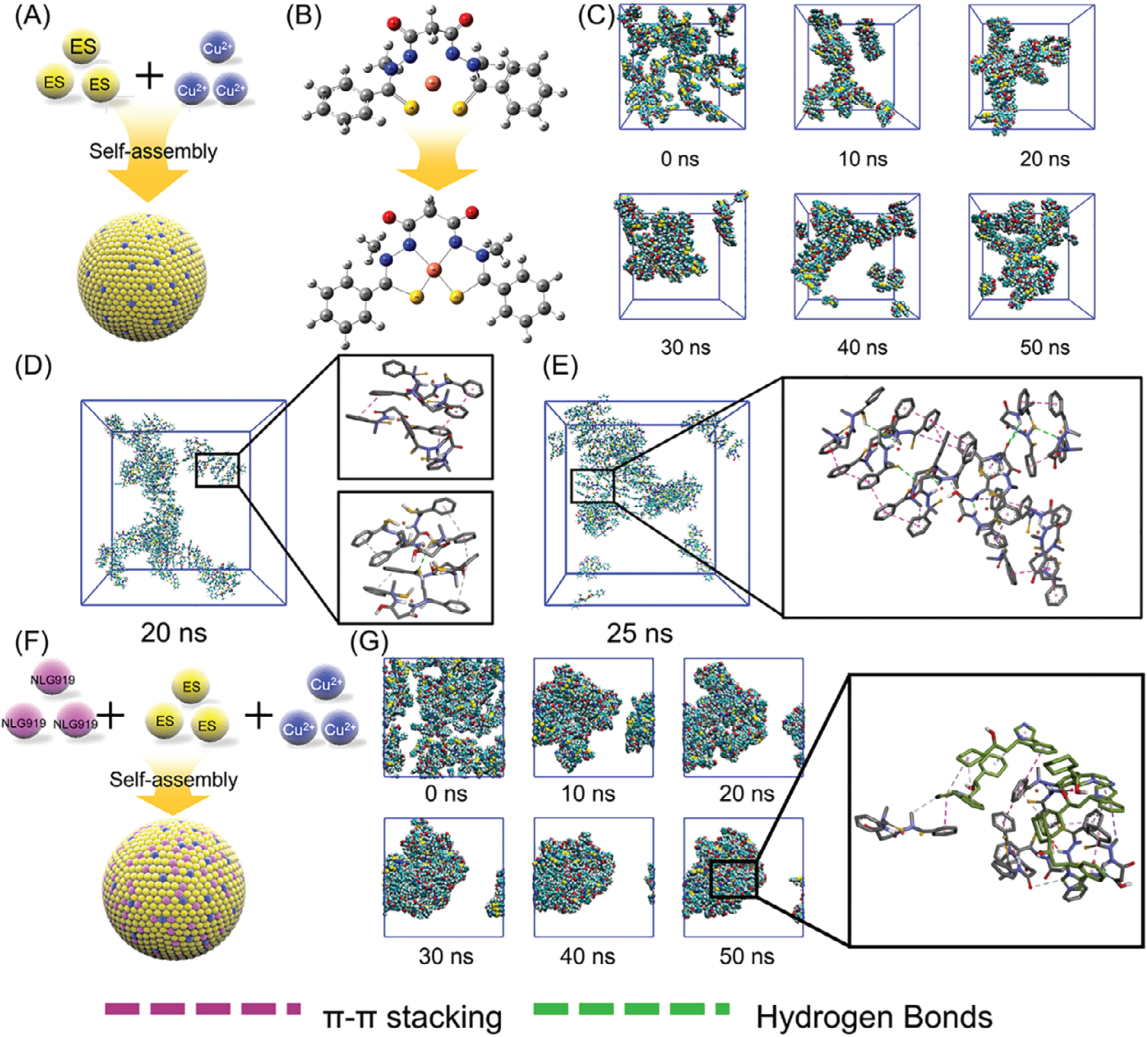
Molecular dynamic simulation assay. A) Schematic illustration of EC self‐assembly process. B) Dynamic optimized model of ES‐Cu monomer. C) Visualization of EC self‐assembly process during 50 ns via molecular dynamic simulation. D,E) Interaction force analysis during the EC self‐assembly process at 20 ns and 25 ns, respectively. Pink line: π‐π stacking force. Greem line: hydrogen bonds. F) Schematic illustration of ECN self‐assembly process. G) Visualization of ECN self‐assembly process during 50 ns via molecular dynamic simulation and driving force analysis at 50 ns.

As shown in **Figure** **2**A, ES and Cu could self‐assemble into uniform nanoparticles.^[^ ^35^, ^36^ ^]^ The structure of the ES‐Cu monomer was optimized using Gromacs. Cu^2+^ could coordinate with the secondary amine group and thioketal group in ES to form a stable structure (Figure 2B). The self‐assembly process was stimulated based on the results above with 100 ES‐Cu monomers. As illustrated in Figure 2C, the ES‐Cu monomer was likely to aggregate to form a layer structure, and the layer could further assemble to keep stable. At 20–30 ns, a significant change was observed in the morphology of EC as micelle structure‐ cluster structure‐ micelle structure transition, indicating this structure was rapidly variable and attempting to integrate into the ultimate form. To verify the potential mechanism of self‐assembly and structure transition, the intramolecular interaction force was analyzed. As shown in Figure 2D, at 20 ns, the major force of the nanoparticle fabrication was the π‐π stacking by ES itself, which could form a layer structure, while the layer structure was assembled via both hydrogen bonds and π‐π stacking to form EC. After the turbulent period, ES tended to be stable and form a spheroid‐like cross‐linking structure at 25 ns (Figure 2E).

Then, the self‐assembly mechanism of ECN was investigated. NLG919, ES and Cu^2+^ could also form uniform nanoparticles (Figure 2F). Compared with EC, the self‐assembly process of ECN was likely to be faster and more stable (Figure 2G). At 10 ns, a uniform spheroid‐like structure has already formed and kept stable from 10 to 50 ns. Similarly, we analyzed the driving force of ECN. NLG919 could interact with ES via π‐π stacking interaction, and the introduction of NLG919 interrupted the self‐assembly of the ES‐Cu layer. In contrast, NLG919 effectively interacted with the ES‐Cu layer to form the uniform nanoparticles. Furthermore, NLG919 also formed hydrogen bonds with ES‐Cu, which further facilitated the self‐assembly process and improved the stability of ECN. Herein, it was concluded that the addition of NLG919 contributed a lot to the self‐assembly of the ES‐Cu monomer by the enhanced interaction force.

As the results above, we were also interested in investigating the drug‐release behavior of EC and ECN. As shown in Figure S2 (Supporting Information), compared with EC, ECN released less ES in 48 h incubation without GSH (EC: 32.6%, ECN: 24.4%), which might be due to the extra interaction force provided by NLG919. However, with the addition of GSH, there was no significant difference between EC and ECN, because GSH could react with Cu^2+^ to disrupt the coordination effect and directly dissociate the ES‐Cu monomer, while the extra π‐π stacking interaction could merely be as the icing on the cake. All these results were consistent with the molecular dynamic simulation assay. As the results above, NLG919 was suggested to improve the stability of the chelates in the system circulation without hindering the ES release in the tumor site. As for ECNM, although the ES release ratio was significantly lower than EC, the decorated membrane could detach via the homotypic membrane fusion internalization process. Herein, ECNM could mainly retain the superior stability in system circulation and ignore the release defect when it is applied in vivo. Similarly, the interaction force also directed the storage stability of the self‐assembly nanoparticles.^[^ ^37^, ^38^ ^]^ As the interaction force increased, it was deduced that fewer ES‐Cu monomers tend to detach and reaggregation in ECN compared with EC, thereby increasing the storage stability.

The abovementioned results demonstrated the successful fabrication of the nanoparticles and illustrated the mechanism of self‐assembly and the potential benefit of NLG919 on drug release and storage stability, which provided powerful evidence for further in vitro/*vivo* studies. In summary, ECNM was characterized as facile fabrication, well stability, high drug loading and selective drug release. All these advantages reflected a great potential for clinical application.

### 2.3 In Vitro Antitumor Activity

**Figure 3:**
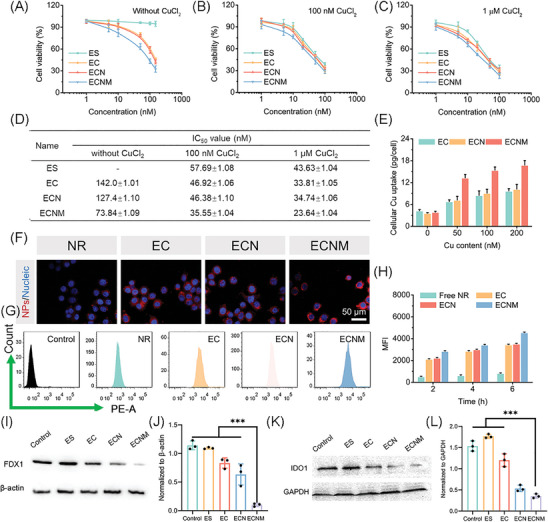
In vitro antitumor activity. A–C) In vitro cytotoxicity of the nanoparticles with different CuCl_2_ concentrations detected by MTT assay, *n* = 6. CuCl_2_ concentration: 0, 100 nM, 1µM. D) IC_50_ value of the nanoparticles against the 4T1 cell line. (E) Intracellular Cu content, *n* = 6. F) Cellular uptake capability of the NR‐loaded nanoparticles observed by CLSM. Red fluorescence: nanoparticles, blue fluorescence: nucleic. Scale bar: 50 µm. G,H) FCM assay and quantitative analysis of cellular uptake, *n* = 6. I,J) FDX1 expression measured by Western blot and the semi‐quantitative analysis. K,L) IDO1 expression measured by Western blot and the semi‐quantitative analysis. Data are presented as mean ± SD. “*”, “**” and “***” represented *p* < 0.05, *p* < 0.01 and *p* < 0.001, respectively.

MTT assay was investigated to evaluate the antitumor activity of the nanoparticles against the 4T1 cell line. As ES is a copper ionophore and intracellular Cu content largely determines the cell viability,^[^ ^7^, ^39^ ^]^ the MTT assay was performed with mediums containing CuCl_2_ with gradient concentration. First, to exclude the influence of CuCl_2_ on cell viability, the cytotoxicity of CuCl_2_ was measured, and no obvious cytotoxicity was observed under the involved concentrations (Figure S3, Supporting Information). Then, the antitumor activity of the nanoparticles was discussed. As shown in **Figure** **3**A–D, ES displayed significant cytotoxicity against the 4T1 cell line with the addition of CuCl_2_, indicating that ES enhanced the cell internalization of Cu and induced Cu‐dependent cell death. Compared with the ES group, EC and ECN gained improved antitumor activity due to the initial Cu loading, while ECNM displayed the strongest toxicity against the 4T1 cell line with the homologous targeting capability by membrane decoration. Moreover, NLG919 also enhanced the antitumor activity of ECN compared with EC, while the effect was masked as the addition of Cu^2+^ (Figure 3D).

Subsequently, the Cu transport capability was investigated with a varied Cu feeding content. As shown in Figure 3E, EC, ECN and ECNM all displayed a concentration‐dependent intracellular Cu transport feature. Among the groups, ECNM gained the highest intracellular Cu content, suggesting that ECNM not only increased the ECN internalization but also freely released ES to transport the supplemental Cu. This might be due to its homotypic membrane fusion internalization pathway. Then, confocal laser scanning microscopy (CLSM) and flow cytometry (FCM) were accessed to evaluate the relationship among cell cytotoxicity, intracellular Cu content and cellular uptake capability. As shown in Figure 3F, ECNM showed the strongest red fluorescence among other reference groups, proving its excellent cellular uptake. FCM was consistent with the CLSM assay (Figure 3G,H). Herein, it is suggested that the homotypic affinity between ECNM and the originating 4T1 cells played an important role in antitumor activity.

Then, we evaluated the cuproptosis induction and TIM regulation potential of ECNM. Cuproptosis, as an emerging cell death pathway, has been proven to regulate FDX1 and dihydrolipoamide S‐acetyltransferase (DLAT), which induces lipoylation and proteotoxic stress. To this end, FDX1 and DLAT expression were both measured. As shown in Figure 3I,J and S4, ECNM could reduce the expression of FDX1 and DLAT, implying sufficient cuproptosis‐inducing capability. NLG919 can inhibit IDO1 expression to regulate Trp/Kyn homeostasis and avoid Trp exhaustion, in turn activating T lymphocytes for cancer immunotherapy. Furthermore, the IDO1 level of nanoparticles‐treated 4T1 tumor cells was measured by Western blot. As shown in Figure 3K,L, ECNM significantly inhibited the expression of IDO1, indicating that ECNM could deliver NLG919 into the cytoplasm and potentially reverse the TIM.

### 2.4 ECNM‐Induced ICD for Cancer Immunotherapy

**Figure 4:**
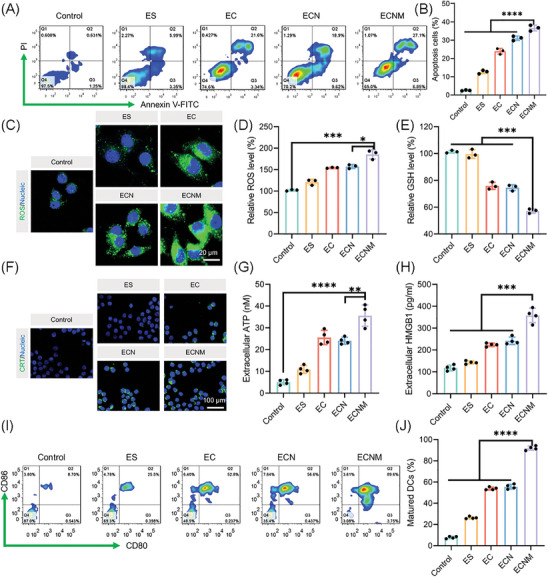
ICD induced by ECNM in vitro. A,B) Representative FCM profiles of the cell apoptosis percentage and the semi‐quantitative analysis. C) CLSM observation of intracellular ROS generation in nanoparticle‐treated 4T1 cell line visualized by DCFH‐DA. Scale bar: 20 µm. D,E) In vitro ROS and GSH level. F) CLSM observation of CRT expression. Scale bar: 100 µm. G,H) Extracellular ATP and HMGB1 level measurement. I,J) The percentage of matured DCs measured by FCM. Data are presented as mean ± SD. “*”, “**” and “***” represented *p* < 0.05, *p* < 0.01 and *p* < 0.001, respectively.

The intracellular GSH level of tumor cells is 10 to 1000‐fold higher than that of normal cells, which reduces Cu^2+^ to Cu^+^. The reduced Cu^+^ could react with H_2_O_2_ to generate highly toxic ·OH, as a Fenton‐like reaction, eventually possessing CDT for tumor killing. To this end, the cell apoptosis assay was applied. As shown in **Figure** **4**A,B, the apoptosis cell percentage of the ECNM group was 2.9‐fold higher than that of the ES group, suggesting that ECNM could effectively trigger tumor cell apoptosis, because Cu^2+^ could not only interact with GSH for GSH consumption but also produce ·OH for ROS generation, synergistically broking the high‐level redox homeostasis of tumor cells. Subsequently, the intracellular GSH and ROS levels were both measured. As shown in Figure 4C, strong green fluorescence was visualized in the ECNM group, confirming that ECNM could effectively produce ROS. Furthermore, ROS and GSH levels were quantitatively analyzed, and ECNM was proven to break the redox homeostasis (Figure 4D,E) and cause oxidative stress for subsequent tumor killing.

As sufficient ROS production could induce ICD and activate antitumor immunity, hallmarks of ICD were systematically analyzed, such as CRT exposure, HMGB1 and ATP release, termed DAMPs. As shown in Figure 4F–H, compared with other groups, ECNM significantly enhanced CRT exposure (strongest green fluorescence in CLSM) and elevated the extracellular ATP and HMGB1 concentration, implying that ECNM effectively induced ICD cascade for cancer immunotherapy. Once ICD is triggered, DAMPs can serve as an “eat me” signal to stimulate DC maturation for antigen‐presenting. The downstream indicators were also evaluated. As shown in Figure 4I,J, ECNM achieved ∼90% matured DCs, which was about ∼1.7‐fold higher than that of ECN and EC.

All these results verified the hypothesis that ECNM could induce cuproptosis/CDT and ICD, which further increased the immunogenicity of tumor cells and activated the antitumor immune response.

### 2.5 In Vivo Biodistribution and Antitumor Efficacy

**Figure 5:**
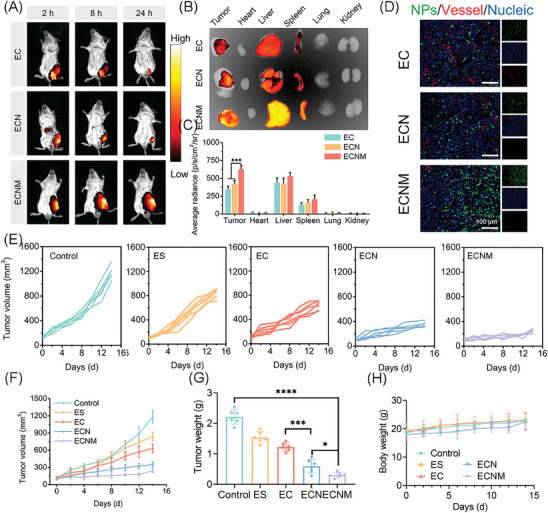
In vivo biodistribution and antitumor activity of ECNM. A) In vivo biodistribution of the nanoparticles in tumor‐bearing mice model. B,C) Ex vivo biodistribution and semi‐quantitative analysis of the nanoparticles in tumors and major organs at 24 h, *n* = 3. D) Intertumoral nanoparticles distribution observed by CLSM. Scale bar: 100 µm. E,F) Individual tumor volume and average tumor volume of the mice treated with the nanoparticles. G) Tumor weight of the mice on the 14^th^ day. I) Body weight of tumor‐bearing mice after treatment. Data are presented as mean ± SD. “*”, “**” and “***” represented *p* < 0.05, *p* < 0.01 and *p* < 0.001, respectively.

The in vivo biodistribution and the tumor accumulation of the antitumor active ingredients greatly determine the therapeutic efficacy. Herein, nanoparticles were labeled with DiR to trace the real‐time in vivo biodistribution behavior. As shown in **Figure** **5**A–C, compared with EC and ECN, ECNM achieved the strongest fluorescence intensity in the tumor region, indicating prolonged tumor retention time and enhanced tumor accumulation. For *ex vivo* bioimaging, ECNM displayed a 1.79‐fold increase in fluorescence intensity than that of EC within tumor tissue. This was due to the enhanced systemic circulation stability and homotypic affinity by 4T1 cell membrane‐coating. It is worth noting that, ECN also seemed to perform better than EC (1.22‐fold), benefiting from the ameliorated system circulation stability via the addition of NLG919 which could enhance the interaction force and reduce the non‐specific dissociation of ECN. Subsequently, DiO was encapsulated into the nanoparticles to visualize the intertumoral biodistribution (Figure 5D). All the above results demonstrated the superior tumor biodistribution of ECNM.

Then, the antitumor efficacy of the nanoparticles was investigated with 4T1 tumor‐bearing Balb/c mice. The mice were randomly divided into five groups and treated with saline (control), ES, EC, ECN and ECNM, respectively. As shown in Figure 5E–G, during the 14‐day treatment, the tumor volume increased rapidly in the control group, and ES was also flat in tumor inhibition due to the typical drawbacks. Compared with the ES group, EC gained enhanced performance with the initial Cu loading, which compensated the intracellular Cu contents and amplified the cuproptosis/CDT process. Then, NLG919 was introduced into the following formulations. ECN displayed higher antitumor efficacy as the presence of NLG919, which could inhibit IDO1 expression to prevent T lymphocyte exhaustion for cancer immunotherapy. As expected, ECNM exhibited the most pronounced antitumor capability, for the reason that ECNM gained superior accumulation in tumor sites and induced cuproptosis/CDT and TIM reversal for synergistic cancer treatment. Meanwhile, a 40‐day monitoring was carried out to investigate the effect of ECNM on prolonging the survival rate of the tumor‐bearing mice. As shown in Figure S5 (Supporting Information), 66.7% of mice for the ECNM group survived in 40 days. In contrast, all mice in the control group died on day 30. All these results demonstrated that ECNM could prolong the survival time of tumor‐bearing mice with better therapeutic outcomes.

Subsequently, the biosafety of the nanoparticles was evaluated via tumor weight and blood biochemistry. As shown in Figure 5I, the body weight was steadily increased in all the groups with no significant difference. Meanwhile, no obvious influence could be observed in ALT, AST and BUN, and no apparent histological damage was found in major organs (heart, liver, spleen, lung and kidney) (Figures S6,S7, Supporting Information). All these results demonstrated that ECNM owed satisfactory biosafety and was suitable for intravenous administration.

### 2.6 ECNM‐Induced Synergistic Immune Response

**Figure 6:**
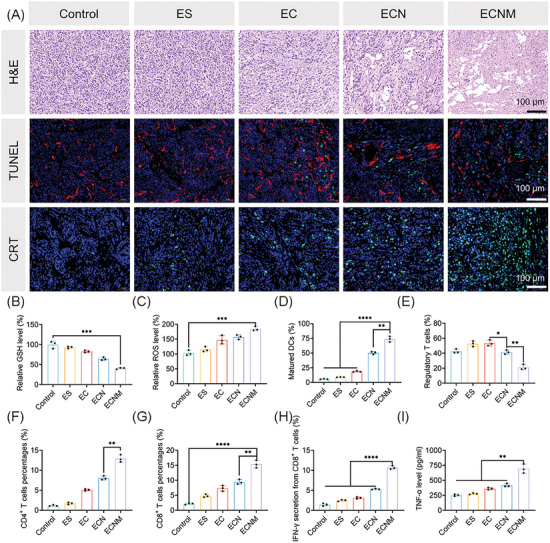
Immune response induced by ECNM in vivo. A) H&E staining, TUNEL immunofluorescence and CRT immunofluorescence assay of tumor tissues. Scale bar: 100 µm. B,C) Relative GSH and ROS level in tumor tissues. D) Matured DC percentages in TDLNs measured by FCM. E) Regulatory T cells, F) CD4^+^ T cells, G) CD8^+^ T cells, and H) IFN‐γ secretion by CD8^+^ T cells in tumor tissues measured by FCM. I) Serum TNF‐α level measured by ELISA. Data are presented as mean ± SD. “*”, “**” and “***” represented *p* < 0.05, *p* < 0.01 and *p* < 0.001, respectively.

Encouraged by the outstanding antitumor activity of ECNM, the potential synergistic immune mechanism was further investigated in vivo. Tumor tissues were harvested for H&E staining and TUNEL staining. As shown in **Figure** **6**A, for the H&E staining assay, cytoplasm loss, nucleus shrinkage and reduced nucleus density were ubiquity in the ECNM group. Meanwhile, ECNM elevated the TUNEL‐positive cell rate. The above results indicated that ECNM could destroy the integrity of tumor tissues and inactivate the tumor cells. FDX1 immunofluorescence staining was also applied in vivo, the results showing that ECNM did induce cuproptosis (Figure S8, Supporting Information). Then, GSH and ROS levels were measured at the histological level. It showed that ECNM indeed depleted GSH and generated ROS in vivo (Figure 6B,C), finally breaking the intracellular redox homeostasis. The CRT translocation was also visualized by immunofluorescence. The brightest CRT‐related green fluorescence was captured in ECNM‐treated 4T1 tumor cells compared with EC and ECN groups (Figure 6A), proving that ECNM could trigger CRT translocation and induce the ICD cascade. Subsequently, the proliferation and maturation of DC as the downstream signal were evaluated. Tumor‐draining lymph nodes (TDLN) were harvested and prepared into the single‐cell suspension, and the matured DC percentage was measured by FCM. As shown in Figure 6D and Figure S9 (Supporting Information), the matured DC percentage was improved by ECNM with nearly 3.96 and 1.46 times higher than that of EC and ECN, respectively, indicating that ECNM induced DC maturation for antigen‐presenting and activating T lymphocyte proliferation. However, as is known to all, immunotherapy is commonly suppressed by TIM in most of the tumor treatments. The upregulation of IDO1 inhibited cytotoxic T lymphocyte activity and promoted its apoptosis. Meanwhile, IDO1 upregulation also consumed Kyn and increased Trp for T_regs_ recruitment and proliferation. Therefore, to investigate whether ECNM could reserve TIM in vivo, the percentage of T_regs_ as the direct evidence was measured. As shown in Figure 6E and Figure S10 (Supporting Information), ECN and ECNM effectively inhibited T_regs_ recruitment and proliferation benefiting from the co‐delivery of NLG919. As for ECN, although the addition of NLG919 had no inherent ICD‐induced capacity (Figure 4F–H), it was deduced to enhance tumor regression by reversing TIM and lead following improvement of tumor permeability and drug accumulation, acting as a positive feedback loop for amplifying ICD and immune response.^[^ ^40^, ^41^ ^]^ Naturally, the superiority of ECNM was due to the cooperation of NLG919 and membrane decoration.

T lymphocyte infiltration positively correlates with the therapeutic efficacy of cancer immunotherapy because of IFN‐γ production. Therefore, CD4^+^ T cells, CD8^+^ T cells and IFN‐γ secretion from CD8^+^ T cells were all measured. Compared with the control group, the frequency of CD4^+^ (Figure 6F; Figure S11, Supporting Information) and CD8^+^ T cells (Figure 6G) were both increased by ECNM, and IFN‐γ secretion from CD8^+^ T cells (Figure 6H; Figure S12, Supporting Information) was also significantly elevated. Finally, serum TNF‐α level was measured by ELISA assay. As shown in Figure 6I, ECNM enhanced the serum TNF‐α level and activated the antitumor immune response. In summary, integrating cuproptosis/CDT and reversing TIM is a promising immunotherapy strategy for cancer management.

### 2.7 Abscopal Effect and In Vivo Vaccination Assay

**Figure 7:**
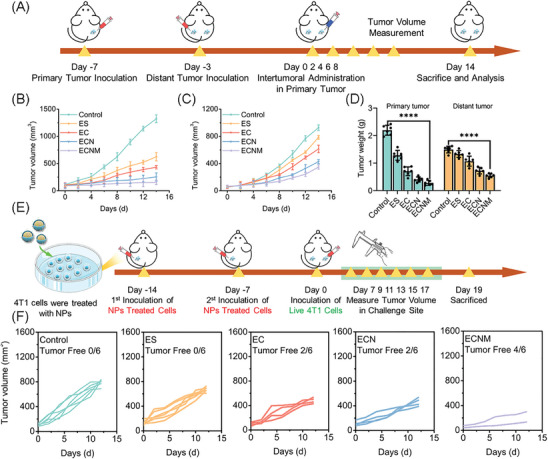
Abscopal effect and in vivo vaccination assay of ECNM. A) Schematic illustration of the treatment against bilateral tumor model. B,C) Average tumor volume of the primary tumor and distant tumor, respectively. D) Tumor weight of the primary tumor and distant tumor. E) Schematic illustration of in vivo vaccination assay. F) Individual tumor volume of challenge site and number of tumor‐free mice. Data are presented as mean ± SD, *n* = 6. “*”, “**” and “***” represented *p* < 0.05, *p* < 0.01 and *p* < 0.001, respectively.

To further confirm the effect of ECNM on activating the systematic antitumor immune response, a bilateral tumor model was established. As shown in **Figure** **7**A, the primary tumor was inoculated on day ‐7, and an equal amount of tumor cells were inoculated on day ‐3 on the opposite side to prepare a bilateral tumor model. Subsequently, saline (control), ES, EC, ECN and ECNM were intratumorally injected into the primary sites at day 0, 2, 4, 6, 8, 10. The tumor volume of both sites was measured and calculated. As shown in Figure 7B, compared with intravenous administration (Figure 5F), intratumorally injection of the nanoparticles in the primary tumor gained a higher tumor inhibition rate due to the suddenly enhanced drug retention. Unlike the primary tumor, the therapy of distant tumors was mostly ascribed to systematic antitumor immunity. Among the groups, ECNM could effectively inhibit both primary and distant tumors (Figure 7C,D). Then, histological analysis of the distant tumors was applied. ECNM was proven to promote DC maturation in the TDLNs (Figures S13,S14, Supporting Information) and CD8^+^ T cell infiltration and downregulate the Ki67 expression in the distant tumors (Figures S15,S16, Supporting Information). Meanwhile, ECNM could effectively increase pro‐inflammatory cytokines levels, such as IFN‐γ and TNF‐α (Figures S17,S18, Supporting Information). All the above results underscored that ECNM successfully induced tumor cell death via the ICD pathway and activated systematic antitumor immune response to inhibit distant tumor progression.

Whole cell‐based vaccination in immunocompetent animals is the golden standard for confirming the ICD cascade. Therefore, an in vivo vaccination assay was investigated. As shown in Figure 7E, 4T1 cells were pretreated with the nanoparticles and subcutaneously administrated into the flank of the mice as a “cell vaccine”. Subsequently, an equal amount of living tumor cells was administrated into the opposite side of the mice, and the tumor volume was recorded. As shown in Figure 7F, the mice with saline‐pretreated tumor cells did not elicit any protective response against the challenge site. In comparison, EC and ECN could significantly inhibit the challenge site tumor growth and partly avoid tumor recurrences. All these results indicated that EC and ECN could induce the ICD cascade and make the tumor cells become a “cancer vaccine”. However, unlike the results of unilateral and bilateral tumor models, there was no difference between EC and ECN in the “cancer vaccine” evaluation. This was because NLG919 had no inherent ICD‐induced capacity, and the trace amount of ECN derived from pre‐treated cells was completely metabolized during the treatment process. Furthermore, with 4T1 tumor cell membrane decoration, ECNM displayed the strongest “vaccination” effect due to sufficient intracellular uptake during pre‐treated. Finally, the mice were sacrificed, and the spleens and tumors in challenge sites were obtained for further investigation. Memory T cells (T_memory_) in the spleen are crucial for the immune memory protection effect and positively correlative to tumor prognosis. As shown in Figures S19,S20 (Supporting Information), ECNM effectively increased the frequency of T_memory_ cells, indicating that ECNM could strongly induce the systematic immune response and immune memory. Meanwhile, CD8^+^ T cell infiltration and H&E staining against distant tumors were investigated (Figure S21, Supporting Information), and results showed that ECNM effectively induced CD8^+^ T lymphocyte infiltration and killed the tumor cells.

## 3 Conclusion

In conclusion, ECNM, a biomimic nanodrug of 4T1 cell membrane‐camouflaged ES/Cu^2+^/NLG919 self‐assembly chelate, was fabricated for cuproptosis/CDT‐induced immunotherapy. ECNM enhanced the systemic circulation stability and the tumor accumulation of ES via homologous targeting mediated by the cell membrane, supplemented by the addition of NLG919. After internalization and GSH‐responsive release, ES could transport Cu^2+^ into tumor cells and trigger cuproptosis. The overloaded Cu^2+^ also induced CDT via a Fenton‐like reaction and disrupted the intracellular redox homeostasis. Furthermore, cuproptosis and Fenton‐like reaction generated a robust ICD effect, together with TIM reversing by NLG919, eliciting and optimizing the T cells mediated immune response for cancer immunotherapy. In summary, ECNM achieves promising tumor suppression with satisfied biosafety and provides a new avenue for cancer therapy.

## 4 Experimental Section

### Materials and Reagents

Elesclomol (ES) was supplied by Aladdin Biotechnology (Shanghai, China). NLG919 was bought from Meilun Biotechnology Company (Dalian, China). Cell membrane protein extraction kit was supported by Beyotime Biotechnology (Shanghai, China). 3‐(4,5‐dimethylthiazol‐2‐yl)‐2,5‐diphenyltetrazolium bromide (MTT), Annexin V‐FITC/PI kit, dichlorodihydrofluorescein diacetate (DCFH‐DA), 2‐(4‐aminophenyl)‐1H‐indole‐6‐carboxamidine (DAPI), culture medium and fetal bovine serum (FBS) were all purchased from Servicebio (Wuhan, China). ATP detection assay kit and anti‐calreticulin antibody were purchased from Abcam (USA). GSH detection kit was purchased from KeyGen BioTECH (Nanjing, China). Copper (Cu) assay kit was purchased from Jiancheng Bioengineering Institute (Nanjing, China). Fluorescence‐labeled antibodies were supplied by Aifang Biological (Changsha, China). All other chemical reagents were analytical grade.

### Cells and Animals

4T1 cell line was purchased from Meilun Biotechnology (Dalian, China) and supplemented with 10% fetal bovine serum (FBS) in the presence of 5% CO_2_ atmosphere at 37 °C. Bone marrow‐derived cells were isolated from the tibia of Balb/c mice and polarized to immature DCs by GM‐CSF/IL‐4.^[^ ^19^ ^]^

To establish 4T1 tumor‐bearing Balb/c mice, 4T1 cells were suspended in PBS with a density of 1×10^7^ cells/0.2 mL and subcutaneously administrated into the buttocks of the animals. When the tumor volume reached approximately 100 mm^3^, the mice were randomly divided into several groups for further experiments. All animal experiments were approved by the Institutional Animal Care and Use Committee (IACUC) of China Pharmaceutical University (24‐03‐027).

### Isolation of 4T1 Tumor Cell Membrane

4T1 tumor cell membrane was extracted by membrane protein isolation kit. Briefly, 4T1 cells were collected and suspended in membrane protein extraction buffer solution. Ultrasound probe sonication was used to destroy the integrity of tumor cells and centrifugation was used to collect the cell membrane. The concentration of the obtained cell membrane was measured by BCA protein assay kits.

### Preparation of ECNM

ECN was fabricated first. Briefly, ES and NLG919 were co‐dissolved in DMSO and copper chloride was added into the system. The system was stirred at room temperature for 6 h. Subsequently, the supernatant was added droplet into distilled water with consistent stirring and dialyzed to remove the residual organic solvent. The prepared ECN was concentrated and stored at 4 °C. EC was prepared by the same method in the absence of NLG919. For ECNM fabrication, 4T1 cell membrane fragments were mixed with ECN. The mixture was sonicated under an ice bath. Then, ECNM was collected by centrifugation.

### Characterization of ECNM

The encapsulation efficacy (EE, %) and loading content (LC, %) of ES and NLG919 were measured by high‐performance liquid chromatography (HPLC). Cu content was measured by a Copper (Cu) assay kit. Then, EE % and LC % were calculated.

The particle size and ζ‐potential of the nanoparticles were measured by Malvern Zetasizer (Nano ZS, UK). The morphology of the nanoparticles was observed by transmission electron microscope (TEM, HT7700, Hitachi, Japan).

SDS‐PAGE was used to confirm the cell membrane coating of ECNM. Briefly, 4T1 cell, 4T1 cell membrane and ECNM were heated within the loading buffer and subjected to SDS‐PAGE and underwent electrophoresis. Then, the gel was collected and stained with Coomassie blue. After washing with the destaining solution, the gel was observed by the imaging system. Then, CD44, CD47 and Na^+^/K^+^‐ATPase were all detected by Western blot according to the standard operation procedure to ensure the integrity of the cell membrane.

The storage stability of the nanoparticles was evaluated. In brief, EC, ECN and ECNM were dispersed into PBS, DMEM and 10% FBS and stored at 4 °C. At predetermined time points, the particle size of the samples was measured by Malvern Zetasizer (Nano ZS, UK).

### In Vitro Drug Release

ECNM was added into a dialysis bag (MW: 3500kDa) and placed into a flask with different buffer solutions (100 mL PBS with 0, 5 and 10 mM GSH, respectively). The flasks were placed into a shaking incubator (100 rpm, 37 °C). At predetermined time points, 1 mL release medium was collected and 1 mL fresh medium was added. The concentration of NLG919 and ES was measured by HPLC.

### Molecular Dynamics Simulation

According to the reported coordination principle and charges distribution,^[^ ^36^ ^]^ EC was constructed using Gaussian with a 2:1 ratio (ES: Cu^2+^). The dynamics optimization of the complex was conducted using GROMACS 2021. The structure of NLG919 was optimized using ACPYPE. The GROMACS 2021 based on the AMBER‐GAFF force field was used to predict the self‐assembly behavior of 100 elesclomol‐Cu^2+^ complexes and 100 elesclomol‐Cu^2+^‐NLG919 complexes, respectively. The monomers were randomly distributed in a cubic periodic box with a side length of 60 nm using PACKMOL, with the water model set as SPCE and a simulation time step of 2 fs. The cutoff for van der Waals interactions was 1.4 nm. The Particle Mesh Ewald (PME) algorithm was used for long‐range electrostatic interactions with a cutoff of 1.4 nm. All systems underwent 5 × 10^4^ steps of energy minimization. The NVT ensemble used the V‐rescale thermostat to maintain a constant temperature of 298.5 K for a 0.1 ns equilibration. The NPT ensemble used the isotropic Parrinello‐Rahman barostat at 1 bar pressure for a 500 ps simulation at 298.5 K. 3D periodic boundary conditions were employed, and the LINCS algorithm was used to constrain all hydrogen bonds. Subsequently, a 50 ns production was conducted. After the simulation, visualization analysis of the simulation was performed using VMD, PyMOL and Discovery Studio.

### In Vitro Cell Cytotoxicity

The MTT method was used to investigate the cell cytotoxicity of the nanoparticles with or without the presence of CuCl_2_. Briefly, 4T1 tumor cells were seeded into 96‐well plates to allow attachment. Subsequently, the medium was removed and the nanoparticles‐containing culture medium was added. After 24 h incubation, MTT was added for another 4 h. Then, the medium was removed and 100 µL DMSO was applied to dissolve the purple crystal, and the absorbance was measured at 450 nm. Furthermore, the cell cytotoxicity of the nanoparticles was also evaluated in the presence of CuCl_2_. The method was the same as above, despite the medium being replaced by a different CuCl_2_‐containing culture medium. The IC_50_ value was measured by GraphPad Prism 8.0 software.

### Intracellular Cu Content

Intracellular copper concentration was measured using a Copper (Cu) colorimetric assay kit. Briefly, 4T1 tumor cells were seeded into 12‐well plates. To investigate the Cu transport capability, gradient CuCl_2_ was added into the culture medium, and the nanoparticles were added and incubated for 6 h. Then, the plate was washed with PBS, and the intracellular Cu concentration was measured with a Copper (Cu) colorimetric assay kit.

### In Vitro Cellular Uptake Assay

To investigate the cellular uptake of the drug delivery systems, Nile red (NR) was loaded into the nanoparticles as a fluorescence probe. Meanwhile, CLSM and FCM assay were both used to investigate the cellular uptake behavior. For the CLSM assay, 4T1 cells were seeded into glass‐covered 12‐well plates to allow attachment. Then, the culture medium was replaced by the serum‐free medium with NR‐loaded nanoparticles. After incubation, the cells were washed and fixed. Nucleic was stained with Hoechst 33 342, and the slides were observed by CLSM. For the FCM assay, cells were harvested after incubation with the nanoparticles and washing with PBS. The intracellular fluorescence intensity was measured by FCM.

### In Vitro Western Blot Assay

Western blot was used to determine IDO1, FDX1 and DLAT expression after treatment with the nanoparticles. For IDO1 expression, 4T1 cells were seeded into 6‐well plates to allow attachment. The cells were pretreated with IFN‐γ to stimulate IDO1 expression. After incubation with the nanoparticles, the cells were harvested and lysed to collect the proteins. Then, loading buffer was added to the protein sample and heated to 100 °C for 15 min. IDO1 protein was separated by SDS‐PAGE and transferred into the PVDF membrane. The membrane was blocked and incubated with the antibodies and finally observed by the imaging system. FDX1 and DLAT expression were measured by the same method without the IFN‐γ pre‐treatment procedure.

### In Vitro Cell Apoptosis Assay

In vitro cell apoptosis assay was investigated using Annexin V‐FITC kits. Briefly, cells were seeded into 6‐well plates to allow attachment. Subsequently, the nanoparticles were added and incubated for 24 h. The cells were washed with PBS and harvested by trypsin. Then, the cells were resuspended with the staining buffer containing Annexin V‐FITC and PI. The cells were finally rinsed with PBS and measured by FCM.

### Intracellular ROS and GSH Detection

Intracellular ROS and GSH were measured by ROS fluorometric assay kit and GSH colorimetric assay kit, respectively. Briefly, cells were seeded into 6‐well plates to allow attachment. Subsequently, the nanoparticles were added and incubated for 12 h. Then, the ROS and GSH levels were measured according to the protocols of the kits.

Furthermore, DCFH‐DA was also used as a fluorescence probe to detect the ROS level. Briefly, cells were seeded into glass‐covered 6‐well plates to allow attachment. After 6 h incubation with the nanoparticles, the cells were stained with DCFH‐DA and observed by CLSM.

### In Vitro ICD Induction

CRT expression was measured by CLSM. Briefly, cells were seeded into glass‐covered 6‐well plates. After attachment, the nanoparticles were added and incubated for 24 h. Then, the cells were washed and fixed. Finally, the cells were incubated with antibodies to visualize CRT expression and observed by CLSM. For HMGB1 and ATP detection, after incubation with the nanoparticles, the culture medium was collected and the released HMGB1 and extracellular ATP levels were measured by corresponding kits. To evaluate the DC maturation, 4T1 tumor cells were incubated with the nanoparticles to allow internalization. Then, the cells were harvested and co‐incubated with BMDCs for 24 h. Finally, the co‐culture cell model was washed and stained with cocktail antibodies.

### In Vivo Biodistribution Assay

4T1 tumor‐bearing Balb/c mice were randomly divided into three groups including EC, ECN and ECNM. DiR was used as a fluorescence probe to label the nanoparticles. The labeled nanoparticles were intravenously administrated. At interval time points, the mice were anesthetized for in vivo fluorescence imaging. Then, the major organs of the mice were harvested for *ex vivo* fluorescence imaging. Intertumoral biodistribution was also investigated with DiO as a fluorescence dye. The tumor tissues were embedded with OCT for frozen slides. The slides were stained to visualize nuclei and vessels and observed by CLSM.

### In Vivo Antitumor Activity

4T1 tumor‐bearing Balb/c mice were randomly divided into five groups including (control, ES, EC, ECN and ECNM groups, respectively, *n* = 6). When the tumor volume reached approximately 100 mm^3^, the nanoparticles were intravenously administrated via the tail vein every other day for 5 times. The tumor volume and body weight were both measured. On the 14^th^ day, the mice were sacrificed, and the tumors were obtained, weighed and stained (H&E staining, TUNEL immunofluorescence and CRT staining). Meanwhile, the blood samples and major organs were also obtained for blood biochemistry assay and H&E staining, respectively. The survival curves were described.

### In Vivo Cuproptosis Evaluation

Cuproptosis was detected by FDX1 immunofluorescence assay. Briefly, after treatments, the tumor tissues were embedded with OCT for frozen slides. The slides were stained and observed by CLSM.

### In Vivo ROS and GSH Level

Similarly, ROS and GSH levels were also measured by ROS and GSH assay kits according to the protocols of the kits.

### Immune Cells and Cytokines Analysis

The immune cell subtypes were stained by cocktail antibodies and measured by FCM. In brief, the tumor tissues were cut into pieces and digested into a single‐cell suspension. After rinsing with PBS, the cells were re‐suspended into FCN staining buffer for the following studies. For matured DC measurement, TDLNs were collected and prepared as a single‐cell suspension. Subsequently, the sample was stained, and the matured DCs subpopulation was gated as CD11c^+^CD80^+^CD86^+^. T_regs_ were measured using a mouse regulatory T cell Staining kit according to the procedure. T_regs_ subpopulation was gated as CD4^+^CD25^+^Foxp3^+^. Effector T cells and IFN‐γ secreted CD8^+^ T cells were also stained. CD4^+^ T cells were gated as CD3^+^CD4^+^CD8^−^, while CD8^+^ T cells were gated as CD3^+^CD4^−^CD8^+^. IFN‐γ secreted CD8^+^ T cells were gated as CD3^+^CD4^−^CD8^+^IFN‐γ^+^. Tmemory lymphocytes was gated as CD3^+^CD8^+^CD44^high^CD62L^low^. TNF‐α level was measured by mouse TNF‐α ELISA kit. All the operations were applied according to the procedure.

### Abscopal Effect Evaluation

To investigate whether ECNM could induce effective antitumor immunity, a bilateral tumor model was established. On the day ‐7^th^ and day ‐3^rd^, mice received subcutaneous administration of 4T1 tumor cells at the right and left flanks, respectively. Then, the mice were randomly divided into five groups, including control, ES, EC, ECN and ECNM, respectively (*n* = 6). On days 0, 2, 4, 6 and 8, the right‐side tumors were treated with the nanoparticles via intertumoral administration, while the left tumors received no treatment. The tumor volume of both the primary and distant tumors was measured. Finally, the distant tumors were harvested for CD8^+^ T cells and Ki67 detections, relative cytokines level in distant tumors was also measured by ELISA.

### In Vivo Vaccination Study

4T1 tumor cells were treated with PBS, ES, EC, ECN and ECNM for 12 h, respectively, and the tumor cells were washed and collected. Then, the drug‐treated tumor cells were injected into the flanks of healthy Balb/c mice on the days of ‐14^th^ and ‐7^th^. On day 0^th^, living 4T1 tumor cells were subcutaneously administered into the opposite flank of the Balb/c mice. From day 7^th^, the tumor volume on the challenge sites was measured every other day. Meanwhile, the number of tumor‐free mice was also recorded. Finally, tumors on challenge sites were obtained for CD8^+^ T cell immunofluorescence and H&E staining. Meanwhile, the spleen was obtained for flow cytometry to detect the T memory lymphocyte percentage and investigate whether ECNM could display a long‐term antitumor immune response.

### Statistical Analysis

All data were presented as mean ± SD. The significant difference was analyzed by one‐way ANOVA. *P* < 0.05 was considered as significant. * *P* < 0.05, ** *P* < 0.01, *** *P* < 0.001, **** *P* < 0.0001.

## Conflict of Interest

The authors declare no conflict of interest.

## Author Contributions

H.W. and X.L. were responsible for all experiments and the original draft preparation and contribute equally to this work. Y.H., J., Z.Z., Y.L. and Y.Z. were partly investigated in experiments and responsible for statistical data analysis. J.B. was responsible for writing reviews. X.L. and Y.H. were responsible for providing project‐related clinical information. Y.L., H.L., and X.J. were responsible for the experiment design, conceptualization, validation, supervision and financial support.

## Supporting information



Supporting Information

## Data Availability

The data that support the findings of this study are available from the corresponding author upon reasonable request.
