# Supplementary material for: Biomimic Nanodrugs Overcome Tumor Immunosuppressive Microenvironment to Enhance Cuproptosis/Chemodynamic‐Induced Cancer Immunotherapy
Source: Adv Sci (Weinh). 2024 Dec 12;12(5):2411122. doi: 10.1002/advs.202411122 (PMC11791997; doi:10.1002/advs.202411122)
Supplement: Supplementary file 1 — Supporting Information [file ADVS-12-2411122-s001.docx]

*Supporting Information for*

**Biomimic Nanodrugs Overcome Tumor Immunosuppressive Microenvironment to Enhance Cuproptosis/Chemodynamic-Induced Cancer Immunotherapy**

***Hangyi Wu^a,1^, Xiaoyu Lu^b,1^, Yuhan Hu^a^, J.Baatarbolat^a^, Zhihao Zhang^a^, Yiping Liang^a,b,c^, Youwen Zhang^c^, Ye Liu^c,^*********, Huixia Lv^a,^*********, Xin Jin^c,^*********.***

^a^Department of Pharmaceutics, China Pharmaceutical University, Nanjing 211198, Jiangsu, China

^b^Phase I clinical trial center, The Affiliated Suzhou Hospital of Nanjing Medical University, Suzhou 215000, Jiangsu, China.

^c^Department of Pharmaceutics, The affiliated Suqian First People's Hospital of Nanjing Medical University, Suqian 223800, Jiangsu, China.

*Corresponding authors. Tel./fax: +86 18751088009 (Ye Liu); +86 13912965842 (Huixia Lv); +86 15951896676 (Xin Jin).

E-mail addresses: liuyede163youxiang@163.com (Ye Liu); lvhuixia@163.com (Huixia Lv); jinxin871211@163.com (Xin Jin).

**Table S1** The encapsulation efficacy (EE, %) and loading content (LC, %) of the nanoparticles. Data are presented as mean ± SD, *n* = 3.

| Name | EE (%) of ES | EE (%) of NLG919 | LC % of ES | LC % of NLG919 |
| --- | --- | --- | --- | --- |
| EC | 86.9 ± 2.3 | - | 86.5±0.7 | - |
| ECN | 93.4 ± 1.6 | 92.3 ± 4.2 | 46.6 ± 0.9 | 46.0 ± 1.2 |
| ECNM | 92.5 ± 3.8 | 95.5 ± 3.5 | 45.5 ± 1.4 | 47.0 ± 1.2 |


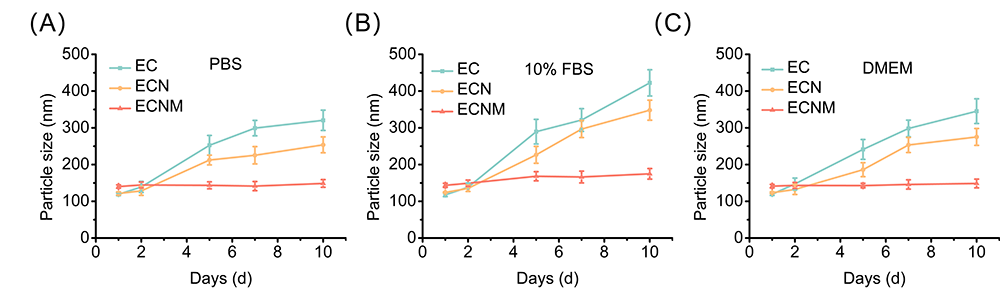


**Figure S1** *In vitro* storage stability of the nanoparticles. Data are presented as mean ± SD, *n* = 3.


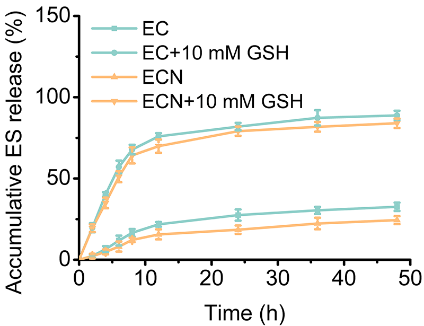


**Figure S2** *In vitro* ES release from EC and ECN. Data are presented as mean ± SD, *n* = 3.


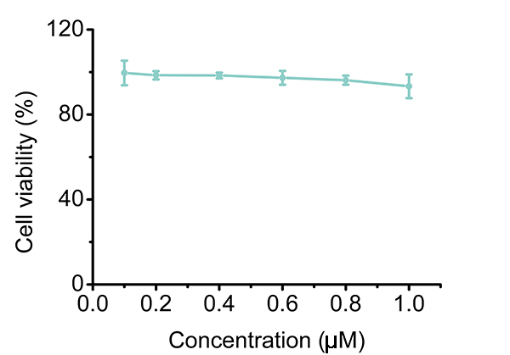


**Figure S3** *In vitro* cytotoxicity of CuCl_2_ against 4T1 cell line. Data are presented as mean ± SD, *n* = 6.


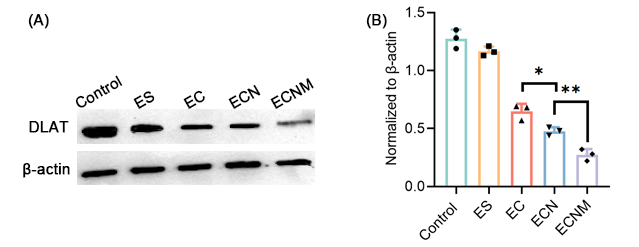


**Figure S4** *In vitro* DLAT expression against 4T1 cell line after treatment with the nanoparticles. Data are presented as mean ± SD, *n* = 3. “*”, “**” and “***” represented *p* < 0.05, *p* < 0.01 and *p* < 0.001, respectively.


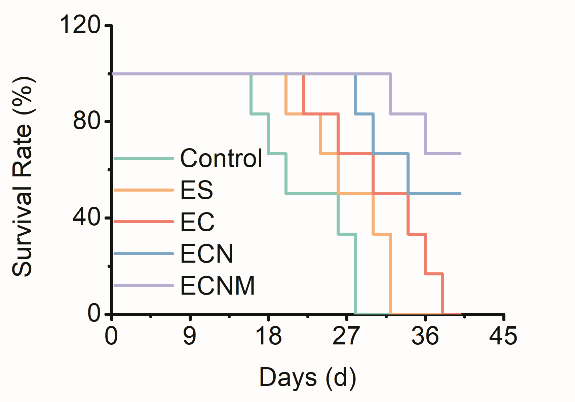


**Figure S5** Survival curves of the treated mice during 40 days.


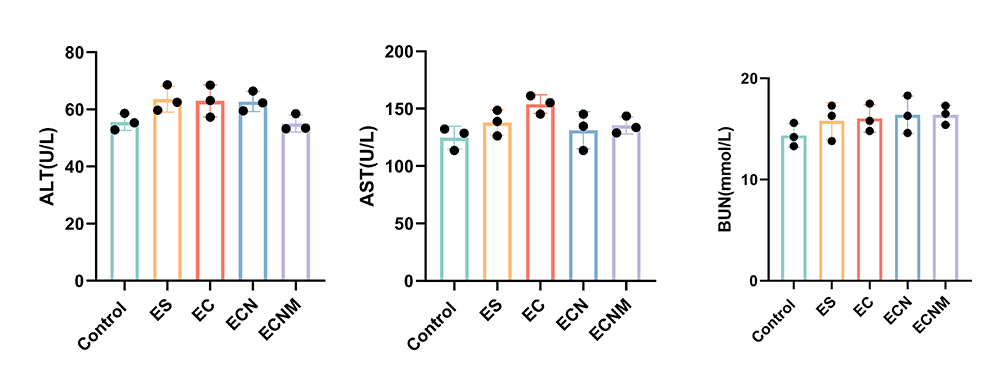


**Figure S6** Serum ALT, AST and BUN level of tumor-bearing mice treated with the nanoparticles.Data are presented as mean ± SD, *n* = 3. “*”, “**” and “***” represented *p* < 0.05, *p* < 0.01 and *p* < 0.001, respectively.


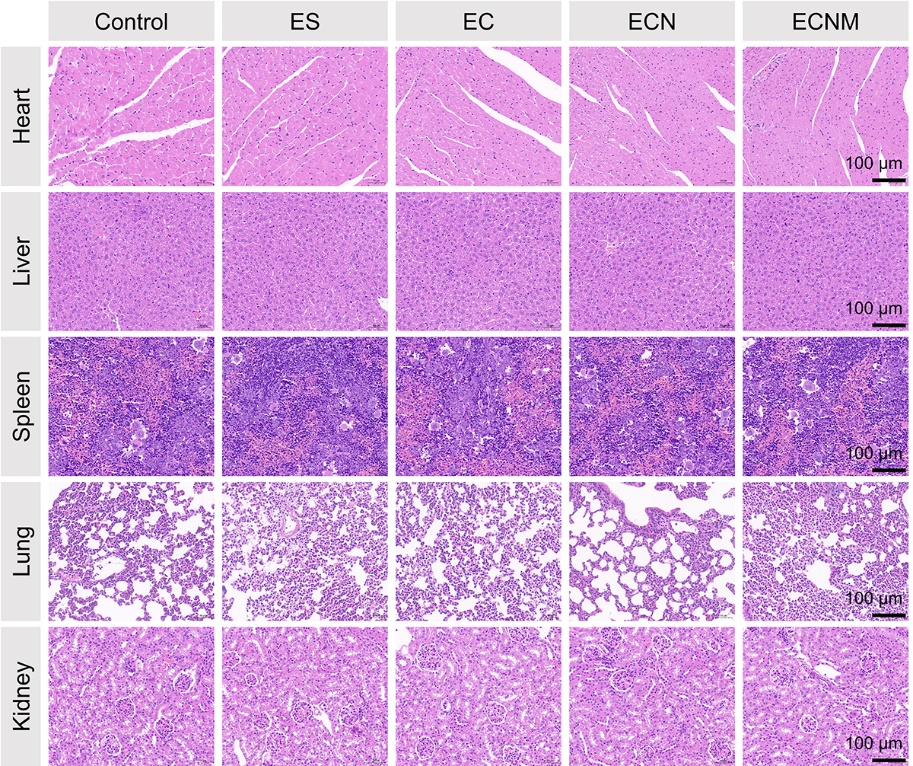


**Figure S7** H&E staining assay of the major organs of the mice. Scale bars: 100 μm.


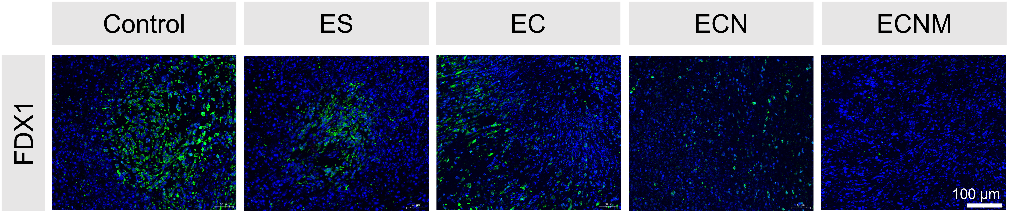


**Figure S8** *In vivo* FDX1 expression observed by CLSM. Green and blue fluorescence represented FDX1 and nucleic, respectively. Scale bar: 100 μm.


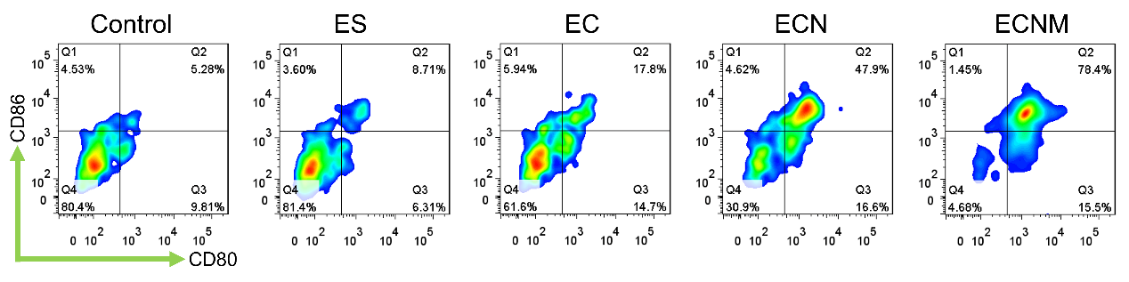


**Figure S9** Matured DC percentages measured by FCM. Matured DCs were gated as CD11c^+^CD80^+^CD86^+^.


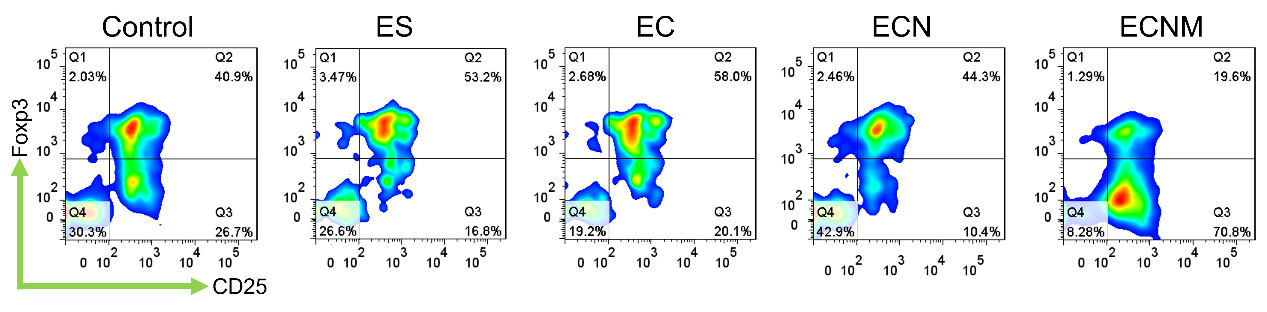


**Figure S10** Regulatory T cell percentages measured by FCN. Regulatory T cells were gated as CD4^+^CD25^+^Foxp3^+^.


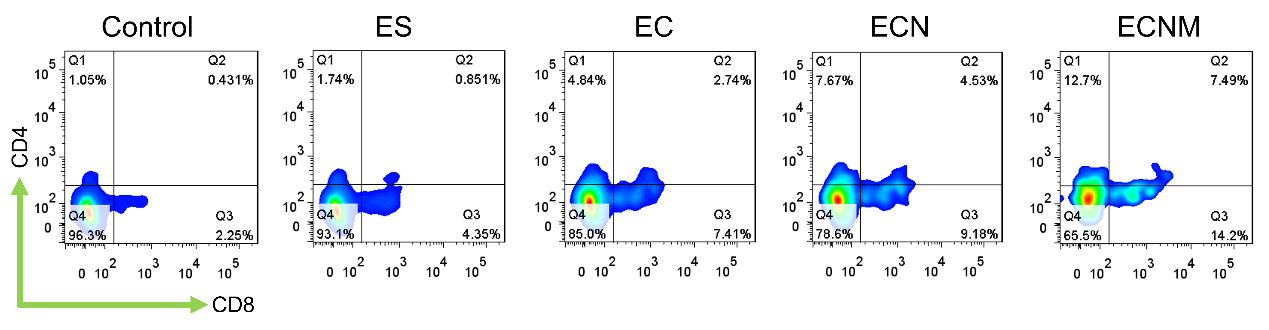


**Figure S11** CD4^+^ and CD8^+^ T cell percentages measured by FCM. CD4^+^ T cells were gated as CD3^+^CD4^+^CD8^-^, CD8^+^ T cells were gated as CD3^+^CD4^-^CD8^+^.


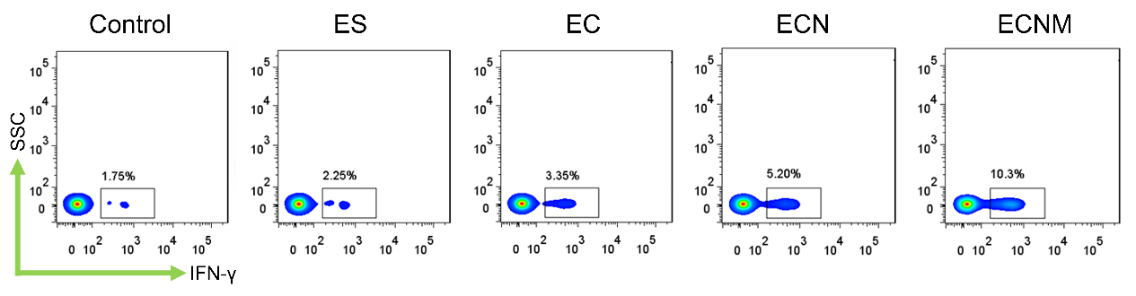


**Figure S12** IFN-γ secretion from CD8^+^ T cell percentages measured by FCM. IFN-γ secretion level by CD8^+^ T cells was gated as CD3^+^CD4^-^CD8^+^ IFN-γ^+^.


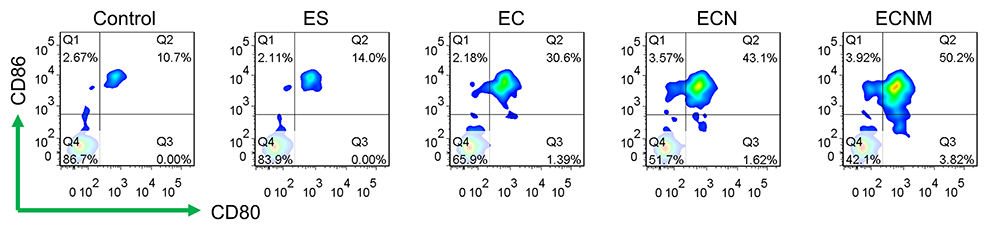


**Figure S13** DC maturation in TDLNs of the distant tumors measured by FCM. Matured DCs were gated as CD11c^+^CD80^+^CD86^+^.


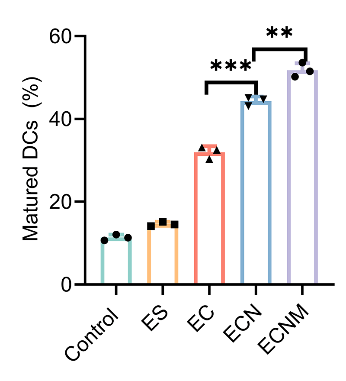


**Figure S14** Histogram analysis of DC maturation in TDLNs of the distant tumors measured by FCM. Data are presented as mean ± SD, *n* = 3. “*”, “**” and “***” represented *p* < 0.05, *p* < 0.01 and *p* < 0.001, respectively.


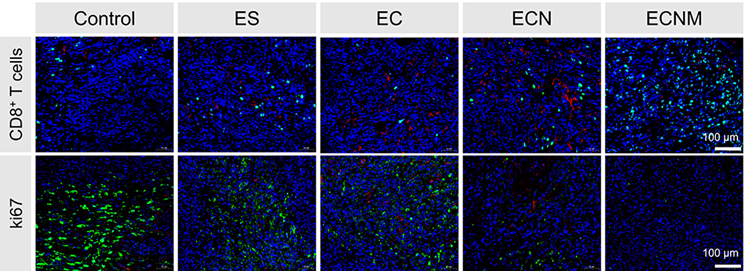


**Figure S15** CD8^+^ T cells and Ki67 immunofluorescence assay from distant tumors. Red, green and blue fluorescence represented vessels, CD8^+^ T cells (or Ki67 positive cells) and nucleic, respectively. Scale bar: 100 μm.

**
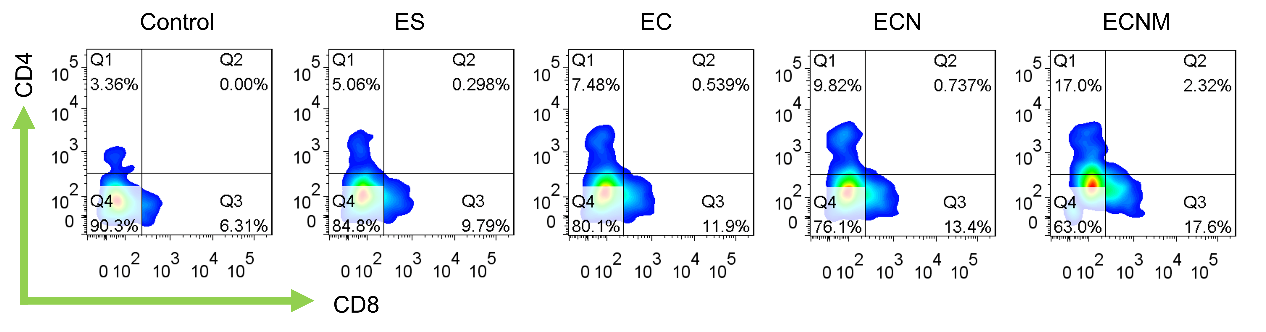
**

**Figure S16** CD8^+^ T cell infiltration in the distant tumors measured by FCM. CD8^+^ T cells were gated as CD3^+^CD4^-^CD8^+^.


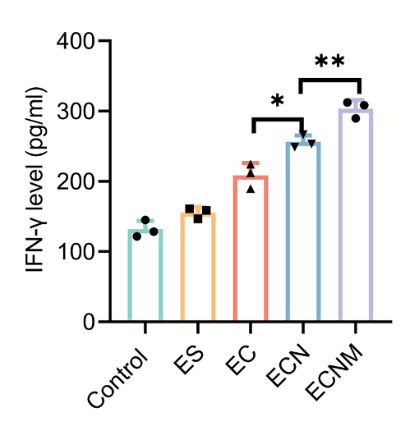


**Figure S17** IFN-γ level in the distant tumors measured by ELISA. Data are presented as mean ± SD, *n* = 3. “*”, “**” and “***” represented *p* < 0.05, *p* < 0.01 and *p* < 0.001, respectively.


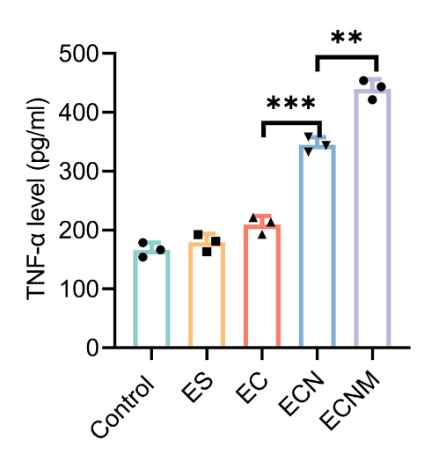


**Figure S18** TNF-α level in the distant tumors measured by ELISA. Data are presented as mean ± SD, *n* = 3. “*”, “**” and “***” represented *p* < 0.05, *p* < 0.01 and *p* < 0.001, respectively.


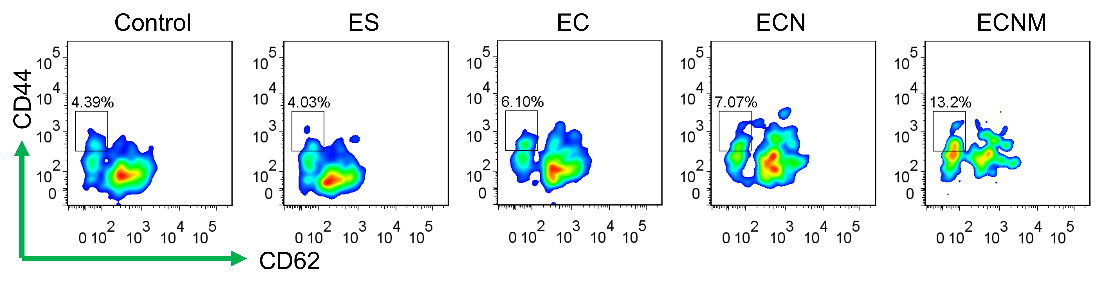


**Figure S19** T_memory_ percentage in spleen measured by FCM. T_memory_ was gated as CD3^+^CD8^+^CD44^high^CD62L^low^.


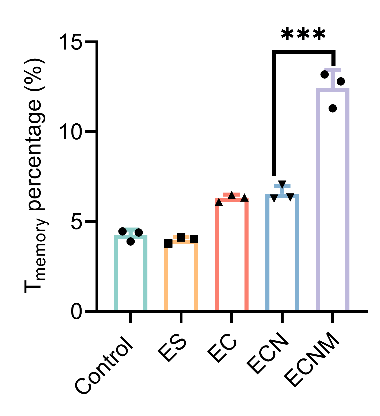


**Figure S20** Histogram analysis of the T_memory_ in the spleen. Data are presented as mean ± SD, *n* = 3. “*”, “**” and “***” represented *p* < 0.05, *p* < 0.01 and *p* < 0.001, respectively.


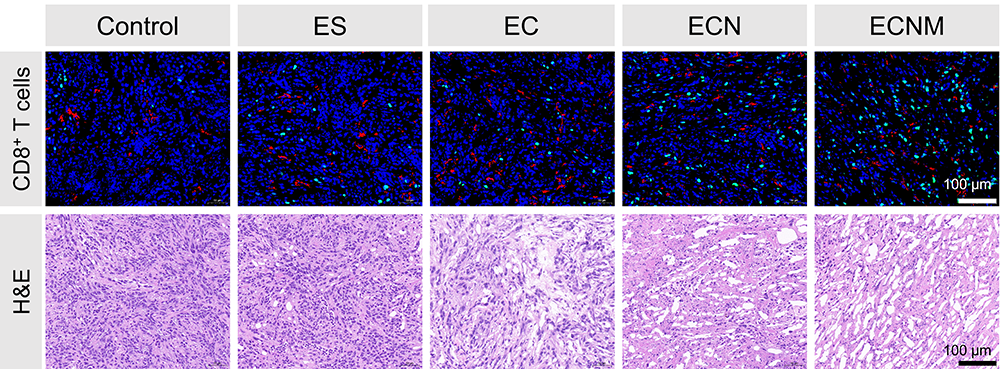


**Figure S21** CD8^+^ T cells immunofluorescence and H&E staining assay from recurrence tumor. Red, green and blue fluorescence represented vessels, CD8^+^ T cells and nucleic, respectively. Scale bar: 100 μm.
